# The complete mitochondrial genome of *Undinula vulgaris* (Dana, 1849) (Crustacea: Calanoida: Calanidae)

**DOI:** 10.1080/23802359.2019.1688724

**Published:** 2019-11-14

**Authors:** Jinwook Back, Hana Kim, Sang-Hwa Lee, Sang-Hui Lee, Myung-Hwa Shin, Byung-Jin Lim

**Affiliations:** Department of Taxonomy and Systematics, National Marine Biodiversity Institute of Korea, Janghang-eup, Republic of Korea

**Keywords:** *Undinula vulgaris*, mitochondrial genome, Calanidae, phylogenetic analysis

## Abstract

The present study reports, for the first time, the complete mitochondrial genome (mitogenome) of *Undinula vulgaris*. The total mitogenome length of *U. vulgaris* was 15,303 bp with 13 protein-coding genes (PCGs), 2 ribosomal RNAs (rRNAs), 22 transfer RNAs (tRNAs), and 1 non-coding region. Phylogenetic analysis showed that *U. vulgaris* belonged to the same family. This is the second report of the complete mitogenome sequence of the family Calanidae.

Species belonging to Calanidae are exclusively marine, with their natural habitat ranging from coastal waters to the open ocean (Boxshall and Halsey 2004). *Undinula vulgaris* is one of the largest calanoid copepods found in tropical and subtropical oceans. They represent important food source for several fish larvae in the northern Gulf of Mexico (Turner 1986). Phylogenetic analysis of the complete mitochondrial genome (mitogenome) is important for gaining insights into the evolutionary relationship of calanoids. However, until now, the mitogenome of only one calanoid (*Calanus hyperboreus*) has been reported. In the present study, we determined the complete mitogenome of *U. vulgaris* for the first time. We believe that this information would be valuable in future phylogenetic studies of calanoid copepods.

Specimens of *U. vulgaris* were collected from Palau, western Pacific Ocean (7°21′49.14″N, 134°23′54.84″E). The voucher specimens were deposited in National Marine Biodiversity Institute of Korea (MABIK Lot no. 0019506). Genomic DNA was isolated from muscle tissue and mitogenome sequences were analyzed on the Illumina HiSeq2000 sequencing platform (Macrogen, Seoul, Korea). Sequences were assembled and annotated using Geneious 10.1.3 (Biomatters Auckland, New Zealand) (Kearse et al. 2012) and the previously reported *C. hyperboreus* mitogenome sequence (Kim et al. 2013) as a reference. The mitogenome annotation server (Bernt et al. 2013) and tRNAscan-SE server (Lowe and Chan 2016) were also used for annotation.

The complete mitogenome of *U. vulagris* (GenBank accession number MN603005) is 15,303 bp in length, containing 13 protein-coding genes (PCGs), 2 ribosomal RNAs (rRNAs), and 22 transfer RNAs (tRNAs) with a non-coding region of 1079 bp. The overall base composition consists of 38.1% A, 10.7% C, 13.2% G, 37.9% T, thereby revealing the high (76%) AT content of the mitogenome.

There were six types of PCG start codons, TAT (nad2-3), TAA (nad4-5), TAC (cytb), ATA (cox2-3, nad6 and ATP8), ATT (cox1and nad1), and ATG (nad4L and ATP6). TAA (nad1, nad6, cox1, cox3, nad4L, ATP6 and ATP8,) ATT (nad2-4 and cytb), and TAG (cox2) were the stop codon, while one (nad5) had an incomplete stop codon, T.

A maximum-likelihood (ML) tree was constructed to confirm the phylogenetic position of *U. vulagaris*, with respect to another three species belonging to the subclass Copepoda. This showed *U. vulgaris* grouped with calanoid species previously announced from GenBank, with high bootstrap values of 100% (Figure 1).

**Figure 1.**
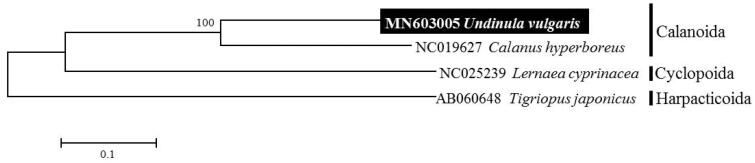
Maximum-likelihood phylogenetic tree based on the 13 protein-coding genes (PCGs) for species of subclass Calanoida. Numbers above the branches indicate ML bootstrap values from 1000 replications.
